# Emerging roles of microfluidics in oral cancer research and clinical translation

**DOI:** 10.1016/j.mtbio.2026.102801

**Published:** 2026-01-13

**Authors:** Zi-Zhan Li, Li-Ya Wei, Lei-Ming Cao, Guang-Rui Wang, Han-Yue Luo, Kan Zhou, Xing-Zhong Zhao, Bing Liu, Ming-Xue Zheng, Chun Xu, Bo Cai, Lin-Lin Bu

**Affiliations:** a State Key Laboratory of Oral & Maxillofacial Reconstruction and Regeneration, Key Laboratory of Oral Biomedicine Ministry of Education, Hubei Key Laboratory of Stomatology, School & Hospital of Stomatology, Department of Oral & Maxillofacial, Head Neck Oncology, Wuhan University, Wuhan, Hubei, 430079, China; b Hubei Key Laboratory of Environmental and Health Effects of Persistent Toxic Substances, School of Environment and Health, Jianghan University, Wuhan, China; c School of Physics and Technology, Wuhan University, Wuhan, China; d Department of Central Laboratory, Jinan Stomatological Hospital, Jinan Key Laboratory of Oral Diseases and Tissue Regeneration & Shandong Provincial Key Medical and Health Laboratory of Oral Diseases and Tissue Regeneration, China; e Sydney Dental School, Faculty of Medicine and Health, The University of Sydney, Camperdown, NSW, 2006, Australia

**Keywords:** Oral cancer, Microfluidics, Liquid biopsy, Sensor, Lab on a chip

## Abstract

Oral cancer remains a global health burden, with limited improvements in long-term survival despite advances in multimodal therapy. Advances in early diagnosis and treatment strategies for oral cancer patients will significantly improve survival outcomes. Microfluidic technology, with its capacity for precise fluid manipulation, high-throughput analysis, and experimental miniaturization, has emerged as a powerful tool to accelerate innovations in cancer research and has become a pivotal pathway in oral cancer investigation and clinical translation. This review systematically examines the expanding roles of microfluidics in oral cancer research, with a particular focus on microfluidics-based liquid biopsy for early detection and prognosis, and microfluidics-enabled therapeutic strategies for treatment development and optimization. By bridging basic research with clinical application, microfluidics holds the potential to revolutionize early diagnosis, precision therapeutics, and functional outcome-oriented management in oral cancer, ultimately improving patient survival and quality of life.

## Graphical abstract

**Figure:**
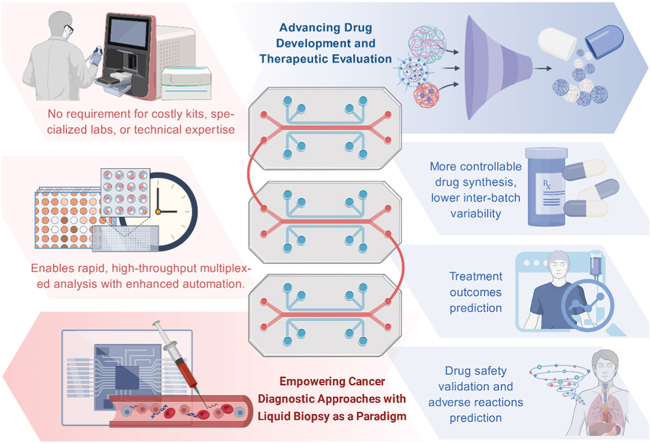

## 1 Introduction

Oral cancer is among the most prevalent malignant tumors of the head and neck, with oral squamous cell carcinoma (OSCC) accounting for approximately 90 % of cases [1]. According to the latest statistics from the National Cancer Center of China, the number of new cases of lip and oral and maxillofacial malignant tumors in China in 2022 was estimated as 65,100, and the number of deaths due to the disease was estimated as 35,200, imposing a substantial public health burden [2]. While surgical resection remains the cornerstone of curative-intent treatment, multimodal therapeutic strategies incorporating radiotherapy, chemotherapy, and immunotherapy have increasingly demonstrated complementary roles in contemporary oral cancer management [[3], [4], [5]]. The synergistic application of multiple therapies has significantly improved the survival of oral cancer patients. However, the five-year survival rate of oral cancer patients remains only around 50 % [6,7]. Paradigm-shifting innovations in early detection methods and therapeutic strategies are therefore critically needed to fundamentally alter the prognostic trajectory of oral cancer patients.

Microfluidics is a technique for manipulating tiny fluid (typically in the nanoliters to picoliters range) in micron-scale channels [8]. Microfluidics was first introduced in 1990 and has been widely used in clinical medicine, chemical analysis, and drug development. The core advantages of microfluidics are its high efficiency, low consumption, high throughput, and precise control, and a single device can perform a variety of laboratory steps, thus enabling the miniaturization and automation of complex experiments [9]. Microfluidic devices are most commonly fabricated from materials such as glass, silicon, polymers, and paper, employing a range of techniques including soft lithography, etching, 3D printing, and nanofabrication [10]. Key design considerations encompass the dimensions and geometry of channels and reservoirs, the types of fluid control elements, and the potential integration of sensors or detection units on-chip [11,12]. These criteria subsequently inform the selection of suitable materials and compatible manufacturing methods. A functional microfluidic system comprises two intrinsically linked components: the chip or substrate that houses the fluidic network, and the actuation scheme or pumping system responsible for mediating and controlling fluid flow [13].

**Fig. 1:**
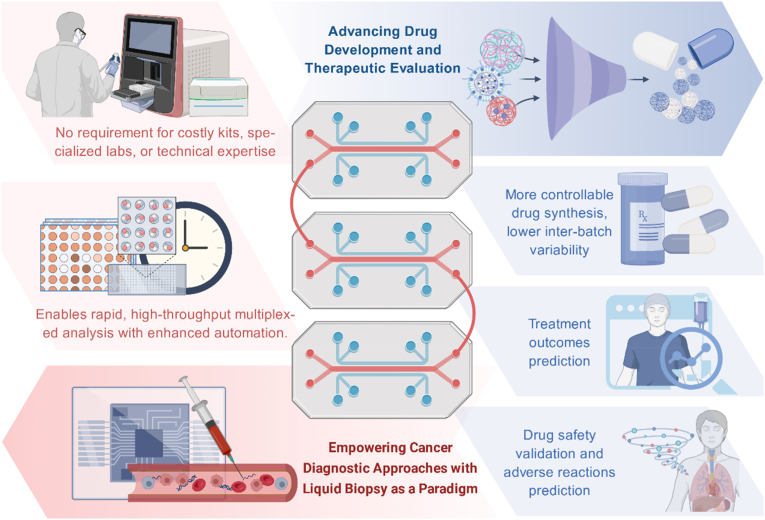
**Current applications of microfluidic technology.** Microfluidic technology serves as a critical platform for liquid biopsy strategies and advances cancer treatment by facilitating drug development, synthesis, efficacy testing, and therapeutic evaluation.

Microfluidics is regarded as a transformative strategy for advancing laboratory testing and clinical practice, showing considerable promise in the fields of disease diagnosis and drug development [14,15]. The rapid processing, cost efficiency, and operational simplicity of microfluidic systems render them highly attractive for detecting analytes in biological specimens [16,17]. This is particularly valuable for rapid diagnosis of acute illnesses and large-scale screening programs addressing major public health concerns [18]. The technology has demonstrated strong potential in monitoring diseases such as breast cancer, prostate cancer, cardiovascular disorders, and SARS-CoV-2 infection [19]. Currently, the application of microfluidic strategy in oral cancer research focuses on drug discovery and development, organoids-on-a-chip and liquid biopsy, and promotes the innovation of early diagnosis and therapeutic strategies for oral cancer (Fig. 1), which has also been mentioned in our previous study [20], and a systematic summarization of microfluidics in oral cancer research will help to promote its clinical translations in this field, thereby improving the clinical care.

This review provides a overview of the expanding applications of microfluidic technology in oral cancer research, spanning fundamental biological investigations, diagnostic innovations, and therapeutic development. This review critically evaluates the advantages, limitations, and specific functions of microfluidic systems across three key domains: tumor organoid modeling for pathobiological studies, pharmaceutical engineering pipelines from drug synthesis to delivery optimization, and liquid biopsy integration for molecular diagnostics. By highlighting emerging trends, such as single-cell analytical platforms, AI-driven microfluidic automation, and multiplexed therapeutic validation systems, this review seeks to connect current technological advances with the challenges of clinical implementation. Ultimately, this critical analysis seeks to catalyze the translation of microfluidic innovations into precision diagnostic tools and personalized treatment strategies, thereby enhancing clinical management paradigms and prognostic outcomes for oral cancer patients.

## 2 Unresolved clinical bottlenecks in oral cancer

The symptoms of early-stage oral cancer are very insidious and most oral cancers are transformed from precancerous lesions such as leukoplakia or epithelial dysplasia, which causes diagnostic difficulties due to the sequential process of its onset and the unclear boundary between precancerous lesions and cancer [21]. For patients with early-stage oral cancer, not only is the treatment process simplified, but the survival benefit is significantly improved. Moreover, in addition to the diagnosis of the disease itself, oral cancer, especially OSCC, is prone to lymph node metastasis (LNM) in the early stage of the disease. LNM is one of the most important indicators of poor prognosis of oral cancer [4,22]. Some studies have shown that the five-year survival rate of OSCC patients without LNM can even be as high as 80 %, whereas it plummets to less than 50 % once LNM occurs [23]. The early onset of LNM is insidious, and the diagnosis of LNM relies heavily on postoperative pathology, which is tied to neck dissection, which leads to a paradox that the diagnosis may occur after the treatment [4], thus there is an urgent need to improve early diagnostic strategies for LNM.

Surgery remains the main treatment strategy for oral cancer, and advances in surgical concepts and the development of precision medicine have significantly improved the prognosis and reduced the incidence of surgical complications [24]. In recent years, adjuvant therapies such as radiotherapy, chemotherapy, immunotherapy and other biological therapies have become important treatment strategies for oral cancer, and the synergism of multiple therapeutic modalities has also improved the inadequacy of monotherapy [25,26]. However, the response to treatment varies from patient to patient owing to the heterogeneity of tumors, which hinders the progress of personalized treatment strategies and causes some uncontrollable treatment complications [27]. The vast majority of patients requiring pharmacological or combination therapy are in locally advanced stages or presenting with LNM (even distant metastases or multiple organ metastases). Reproducibility of treatment for these patients is extremely low, and it is essential to seize the only remaining therapeutic window to administer highly sensitive drugs in order to achieve a survival benefit [28].

**Fig. 2:**
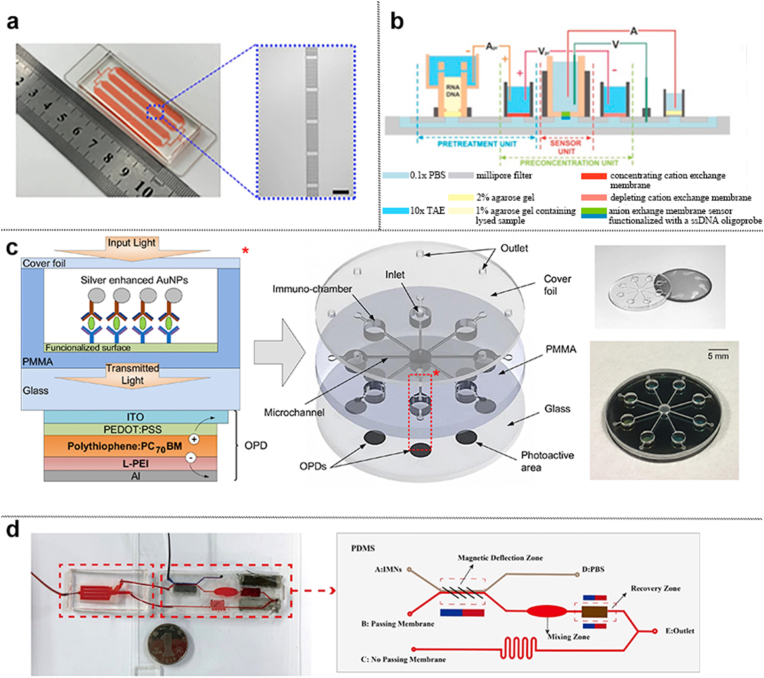
**Multiple designs of microfluidic chips.** a) Microfluidic cascades for extracellular vesicles isolation and purification [20]. b) A detection platform connected by Direct Current [33]. c) Layout of an optical microfluidic biosensor [34]. d) Schematic diagram of the device for extracellular vesicle detection [35]. Panels a–d adapted with permission.

Microfluidics, as an important carrier for liquid biopsy strategies, plays an important role in the early diagnosis or differential diagnosis of oral cancer [29]. Liquid biopsy outperforms conventional imaging methods in the diagnosis of both early-stage cancer and occult LNM, showing significant advantages, including avoidance of radiation exposure and good economic feasibility [30,31]. Recognition and analysis of biomarkers by microfluidic biosensors for early diagnosis of oral cancer or differentiation from precancerous lesions, or early diagnosis of LNM has good clinical translational significance [20] (Fig. 2a). The widely used liquid biopsy modality still relies heavily on enzyme-linked immunosorbent assay (ELISA), but expensive library preparation kits, highly skilled technicians, specialized laboratory setups, and long detection times still limit the clinical application of ELISA [8]. The integration of microfluidics into biosensors helps to enhance their automation capabilities, simplify multiplexing, reduce processing time, and enable high-throughput analysis [32].

Microfluidics contributes to oral cancer treatment in two primary ways: facilitating drug development and nanoparticle synthesis and enabling efficacy testing and treatment evaluation. Specifically, microfluidic platforms support the production of nanodrugs by providing highly controllable nanoparticles (NPs) fabrication with minimal batch-to-batch variability, making them a versatile tool for precise drug development. These NPs-based drugs aim to improve the treatment of oral cancer by addressing challenges such as chemotherapy resistance and potentiating immunotherapy efficacy [36,37].

In addition, microfluidics-based organ-on-chips laboratories offer new ideas for drug development, which includes two aspects. One is that organ-on-chips derived from a patient's tumor may be able to predict the degree of efficacy or therapeutic response of a patient to a drug, which may provide a reference for personalized treatments, such as a patient's response to different chemotherapeutic drugs or to immunotherapy [38,39]. The second is to validate drug safety or make predictions about potential side effects, and in these studies may crosstalk major organs such as the heart, liver, spleen, lungs, and kidneys [40]. The use of multiple organ-on-chips in tandem may allow for multifunctionalization or even simultaneous validation of drug efficacy and safety [41], which will be discussed in detail below.

## 3 Characteristics of microfluidic technology in oral cancer research

The application of microfluidic technology to liquid biopsy in oral cancer primarily involves two approaches: the first integrates microsensors with fluidic components (e.g., pumps and microfluidic chips), while the second focuses on the miniaturization of analytical chemistry methods. The unique advantages of microfluidics are simplicity, portability and miniaturization for real-time analysis, detection and screening for medical conditions [42]. Compared to conventional diagnostics, these microfluidics-based devices are more affordable and do not require expensive large-scale equipment or trained personnel for use in a variety of complex clinical scenarios. In addition, the versatility of microfluidic platforms further enriches their use in oral cancer diagnosis and treatment, and the simultaneous detection of multiple biomarkers may further improve diagnostic accuracy [15]. Thus, the establishment of reliable biomarkers and recent advances in microfluidic biosensing technology can be combined to potentially develop highly sensitive and specific diagnostic tools for the screening and diagnosis of oral cancer.

In addition, microfluidics can also be used for the production of NPs, in which the core advantage of microfluidics is a highly continuous production process with less variation between batches, which may ensure stable efficacy of NPs-based drugs during treatment and may have potential benefits for minimizing drug side effects. When microfluidics is used to predict patient response to therapy or for constructing organ-on-chips, it is possible to construct *in vitro* 3D models that mimic a complex tumor microenvironment (TME) [43,44]. These microfluidic systems can be compartmentalized into interconnected chambers that recreate physiological factors analogous to those in the TME via microfluidic technology, including oxygen concentration, nutrient availability, flow patterns, liquid volume, surface tension, or pressure. These parameters can also be controlled and adjusted, thus allowing for real-time monitoring of very low levels of markers or therapeutic efficacy monitoring mediators from patient-derived cells or tissues, prospectively screening potential candidate therapeutic agents or effective therapeutic concentration ranges for oral cancer, as well as assessing their potential to interact with combinations of other cancer treatment modalities.

## 4 Microfluidics based liquid biopsy of oral cancer

**Fig. 3:**
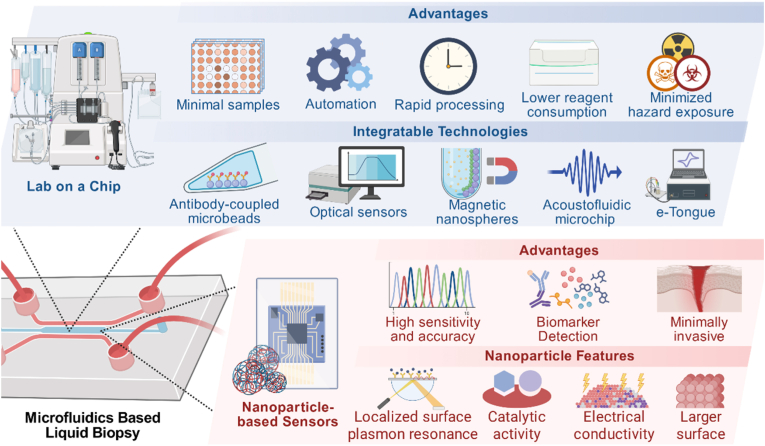
**Microfluidic technology enhances oral cancer diagnosis.** Microfluidic technology leverages two primary forms, lab-on-a-chip and nanoparticle-based sensors, by integrating multiple technologies to enhance oral cancer liquid biopsy.

Owing to their accessibility and cost-efficiency, microfluidic platforms have emerged as valuable tools for liquid biopsy in oral cancer. Fig. 3 and Table 1 illustrate representative microfluidics-based liquid biopsy strategies. Such approaches show potential not only for detecting disease progression at early stages but also for predicting therapeutic response and anticipating adverse effects in patients.

**Table 1:** Applications of microfluidics device in oral cancer research.

Microfluidic device	Biomarkers	Detection threshold	Assay time	Clinical sample	Key advantages	Limitations
A nanostructured microfluidic array [45]	IL-6, IL-8, VEGF, VEGF-C	5 fg mL^−1^ for IL-6, 10 fg mL^−1^ for VEGF and IL-8, and 50 fg mL^−1^ for VEGF-C	50 min	5 μL of serum	Specificity of 98 % for oral cancer; high diagnostic utility, low-cost and easily fabricated	CAlinical sensitivity of 89 %
A microfluidic chip combined with magnetic immunoassay [46]	*anti*-p53	4 ng mL^−1^	60 min	-, salivary	Non-invasive, faster processing and lower limit of detection	Need for reagent concentration optimization, enhanced magnetic control and anti-fouling surface coatings
An optical microfluidic biosensor with polyethylenimine-modified polythiophene-C70 organic photodetectors [34]	IL-8, IL-1β and MMP-8	80–120 pg mL^−1^	30 min	1 μL of salivary	Non-invasive; potential for oral cancer early detection; low detection limits, high detection specificity and reproducibility	Higher estimated concentrations in real saliva because of remaining protein impurities
A coustofluidic Salivary Exosome Isolation [47]	EVs and DNA extracted	47.8 copies of HPV16 DNA mL^−1^	4 h for ddPCR assay turnaround time	-, salivary	Non-invasive; 80 % concordance with tissues positive for HPV16; high-purity and high-yield salivary exosome isolation; single device and automated	–
A magnetic microfluidic device [48]	Annexin V^+^ EGFR^+^ EVs and Annexin V^−^ EGFR^+^ EVs	3.7 × 10^5^ ParticlesmL^−1^ for Annexin V^+^ EGFR ^+^ EVs and 6.0 × 10^5^ particles mL^−1^ for Annexin V^−^ EGFR^+^ EVs	–	-, salivary	Non-invasive, selectively isolating and specifically detecting in complex environments; assisting in distinguishing the patients with early stage; enabling the real-time monitoring of the OSCC progression	–
A microfluidic device-based *in vivo* detection of the PD-L1+ sEVs method [49]	PD-L1^+^ sEVs	3.2 × 10^3^ particles μL^−1^	–	building extracorporeal circulation	Separating quickly and efficiently; automatic precise magnetic field control; monitoring tumor growth, reflecting tumor stage and predicting development trend	
‘Cytology-on-a-chip’ based sensors [50]	cell circularity, Ki67 and EGFR expression, nuclear-cytoplasmic ratio, nuclear area, and cell area	–	20 min	brush cytology samples	Integrating several different sources of information into a successful risk assessment	–
A facile platform containing two microfluidic chips filled with antibody-modified microbeads [51]	ASPH^+^ PDF-EVs	–	Less than 30 min mL^−1^	-, PDF	Utilizing medical waste; enabling fast diagnosis with higher precision or sensitivity	–
A microfluidic microarray capable of lysing cells and quantifying proteins released after lysis [52]	DSG 3	0.10 fg mL^−1^	–	–	The first sub-fg detection level microfluidic device with on-chip lysis and chemiluminescent detection for single cell protein measurement enabling cancer metastasis diagnostics	Limited to the single-cell level
A novel stepwedge microfluidic chip combining magnetic microsphere separation with single-layer fluorescence counting [53]	PD-L1^+^ EVs	850 particles mL^−1^	–	-, salivary	Non-invasive; capable of simultaneously visually and quantitatively detecting EV levels from various cell sources in human body fluids	Relatively narrow capture area for microspheres; high injection pressure; for single type of disease
An AI-derived, non-invasive, label-free 3D-printed microfluidic SERS biosensor platform utilizing Cu@Ag/carbon nanofibers [54]	oral cancer biomarkers such as nitrate, nitrite, thiocyanate, proteins, and amino acids with a micro-molar concentration	10^−12^ M for rhodamine 6G molecules	–	-, salivary	Non-invasive; diagnostic accuracy achieving an overall classification accuracy of 87.5 %, along with specificity of 92 % and sensitivity of 88 %	–
Plasmonic Ag nanocube (AgNC) enhanced SERS biosensing platform [55]	DNA	3.1 fM (S/N = 3)	–	-, salivary	Non-invasive; achieve highly sensitive detection of target DNA	–

IL, interleukin; VEGF, interleukin; MMP, Matrix metalloproteinases; EV, extracellular vesicle; PD-L1, programmed cell death ligand 1; sEV, small extracellular vesicle; PDF, postoperative drainage fluid; DSG, desmoglein.

### 4.1 Microchips

Microchip achieves adaptation, miniaturization, integration, and automation of analytical laboratory workflows within a unified chip-scale platform. Due to the heterogeneous, aggressiveness and susceptibility to early LNM of oral cancer, especially OSCC, early detection and screening of OSCC is a key factor that can significantly improve treatment outcomes and patient survival, and microchip provides a strategy that enables earlier detection of oral cancer compared to radiological or histopathological approaches. The microchip system will accept and process small quantities of biomarkers or biological samples, which then provides numerical values indicating the presence and quantity of specific molecules or substances in the sample, such as pathogen antigens, nucleic acids, antibodies, metabolites, toxins, drugs, and cancer markers. Its unique advantages include small sample size, automation, short processing time, lower reagent consumption, reproducibility and consistency, reduced exposure to hazardous materials or infectious agents, minimal risk of sample contamination, ease of disposal, and inexpensive training of personnel, etc. Microchip is able to bring advanced analytical techniques to underdeveloped and developing countries lacking traditional analytical laboratories to bridge the gap in diagnostic capabilities caused by socio-economic level development. Microchip technology can bring advanced analytical technology to underdeveloped regions and developing countries that lack traditional analytical laboratories, in order to compensate for the differences in diagnostic capabilities caused by the development of socioeconomic levels.

Microchips are categorized by their intended purposes, such as diagnosis, differential diagnosis, treatment guidance, and therapeutic efficacy prediction. Although most oral cancer lesions can be detected by clinical examination, the frequent progression from occult precancerous lesions complicates early detection of oral cancer. Some studies of microchip are limited to the cytological level, i.e., only detecting OSCC cell metabolites or cellular samples such as CTC [56,57], and thus predicting the biological behavior of oral cancer cells. Although these studies have demonstrated certain efficacy, the inherent heterogeneity of oral cancer remains an unresolved challenge. Consequently, current research efforts predominantly focus on optimizing microchip platform performance or validating novel device configurations, while exhibiting limited potential for true clinical translation [33] (Fig. 2b).

**Fig. 4:**
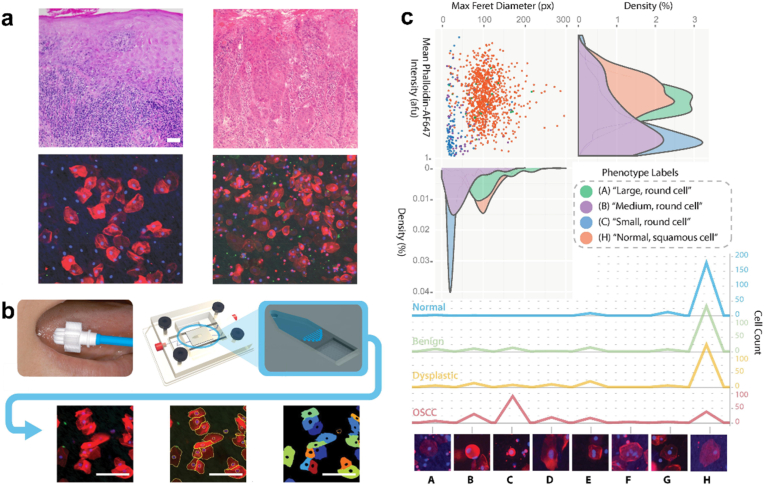
**A microfluidic platform for the detection of oral potentially malignant lesions.** a) Histopathological and immunofluorescence-cytology images of the lesions are shown. b) Schematic diagram of sample processing on the microfluidic chip platform. c) Cellular phenotypes recognized by morphological parameters. Adapted from Abram et al. [50] with permission.

To increase the translational value of these microchips, biological analyses of blood and saliva can detect biomarker levels in different patients, allowing for personalized diagnosis and guided therapy. Using antibody-coupled microbeads integrated into a microfluidic device to construct a microfluidic chip, Ruchika et al. formed a nanostructured microchip by connecting eight similar microchips in series to achieve ultrasensitive simultaneous measurements of IL-6, IL-8, VEGF, and VEGF-C in the serum of 78 patients with oral cancer, and based on a standardized mean of the four proteins measured above, the clinical specificity for the detection of oral cancer based on the standardized average of these four proteins was 98 % and the sensitivity was 89 % [45]. A similar study, also using multiple microfluidic chips based on antibody-coupled microbeads in tandem to form a microchip system, achieved ultrasensitive detection of IL-6 and IL-8 in serum (5 fg/mL and 7 fg/mL) and rapid prediction of oral cancer within less than 30 min [58]. Saliva-based detection using microchip platforms represents an alternative diagnostic strategy. Compared to serum, saliva is part of the oral environment and has superior abundance and content of oral cancer-related biomarkers within it. Lin et al. constructed a microfluidic chip based on antibody-coupled microbeads to biologically detect saliva samples from patients, and analyzed the content of Anti-p53 autoantibodies within the saliva to achieve rapid screening for oral cancer, which shortened the detection time to 1/3 compared to the previous antigen-antibody-reaction method [59]. In addition to the strategy of antibody coupled microbeads, some scholars have also focused on combining optical sensors with microfluidic technology to construct a microchip system to accurately quantify IL-8, IL-1β and MMP-8 in saliva by detecting absorbance utilizing organic photodetectors, which has high detection specificity and short analysis time, and has demonstrated good results and clinical translational potential in differential diagnosis of early oral cancer [34] (Fig. 2c). Similarly, an acoustofluidic microchip was developed to sort and quantify exosomes in saliva to detect HPV-DNA in patients' saliva, with 80 % concordance with pathologic HPV-16 staining, which, although unsatisfactory, provides a faster alternative for diagnosis and prediction of patients with HPV-associated oral cancer [60]. An integrated system of microchip based on magnetic nanospheres is another option, enabling rapid analysis of extracellular vesicles (EVs) in a wide range of biological media for tumor staging prediction and tumor condition detection in OSCC [61,35] (Fig. 2d). Another distinctive class of biomarkers has recently emerged as a compelling diagnostic target. Timothy et al. passed dissociated cell suspensions from tumor tissues of oral cancer patients through a microfluidic device called cytology-on-a-chip to predict the risk and prognosis of different patients and enabled discrimination between oral cancer and precancerous lesions [50] (Fig. 4). The integration of electronic tongue systems with microfluidic technology represents a groundbreaking diagnostic paradigm. Daniel et al. pioneered this approach by coupling a microfluidic e-tongue with hybrid machine learning architectures. The data obtained from the electronic tongue were processed using multidimensional projection techniques and unsupervised and supervised machine learning algorithms. When combined with patients' medical histories and personal adverse habit histories, the approach demonstrated good diagnostic accuracy [62].

**Fig. 5:**
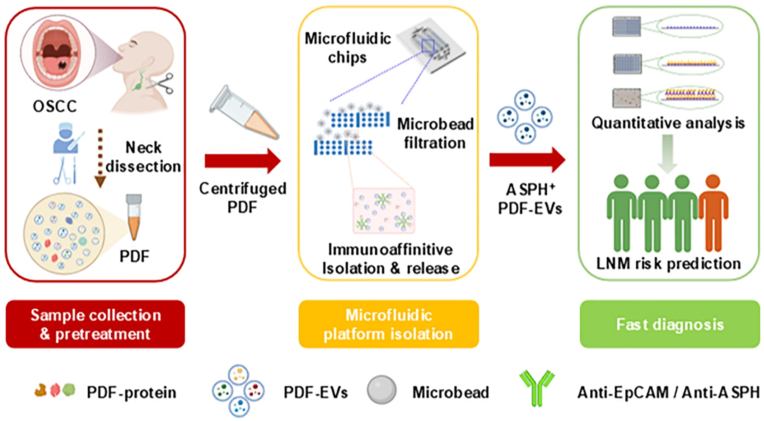
**Schematic of diagnosing LNM in postoperative OSCC patients by PDF-EV analysis based on microfluidic isolation.** Rapid and efficient diagnosis of OSCC postoperative lymph node metastasis by using microfluidic chips to isolate and purify EV in postoperative cervical drainage fluid for ELISA analysis. Adapted from Li et al. [20] with permission.

**Fig. 6:**
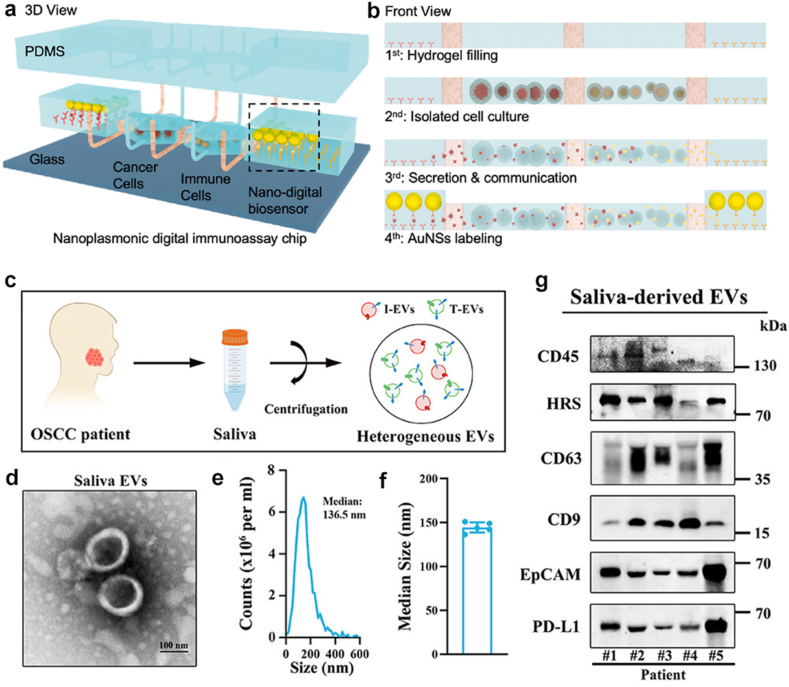
**Detection of PD-L1+ extracellular vesicles using microfluidic chips.** a) 3D schematic of a chip module [64]. b) Workflow of the above module [64]. c)Extracellular vesicles can be collected directly from saliva of oral cancer patients [63]. Characterization of saliva-derived extracellular vesicles using d) transmission electron microscopy [63] and e) nanoparticle tracking analysis [63]. f) The size of saliva-derived extracellular vesicles [63]. g) Western blot result of saliva-derived extracellular vesicles [63]. Panels a–g adapted with permission.

In addition to early diagnosis of oral cancer, the microchip system also plays a role in predicting metastasis or predicting treatment effects. In a previous study, our team designed a microfluidic chip based on antibody-coupled microbeads, and constructed a microchip system through a dual-chip tandem, which accurately and rapidly sorted and analyzed ASPH^+^ EVs from postoperative drainage fluid (PDF) for early prediction of oral cancer LNM, showing excellent sensitivity and good economy [20](Fig. 5). A similar study detected OSCC patient-derived DSG3 to predict the risk of metastasis in OSCC patients [52], However, this study was limited to the single-cell level, focusing on DSG3 expression in cell lysates, which was of limited relevance as a hint for clinical practice. Jia et al. developed a Step-Wedge microfluidic chip based on antibody-coupled microbeads and integrated magnetic nanospheres to sort PD-L1 double-positive EVs in saliva of oral cancer patients to predict the response to immunotherapy and guide the design of treatment regimen [63] (Fig. 6).

### 4.2 Nanoparticle-based sensors

In contrast to microchip, NBS combine NPs with microfluidic devices. NPs are commonly used as components to capture targets and transmit signals due to their localized surface plasmon resonance, catalytic activity, and electrical conductivity, and are often synthesized in situ or simply embedded in a microfluidic system prior to or after addition of analytes. The key step in integrating NPs into microfluidic devices is functionalization modification, and the techniques mainly include thiol modification, EDC-NHS coupling reaction, avidin-biotin conjugation, and π-π interaction [8].

Hema et al. integrated cerium oxide NP into a microfluidic chip to enhance the electrochemical biosensing signal and sensitivity of the biosensor with the help of its high catalytic performance and large surface to achieve highly sensitive detection of IL-8 in saliva for prediction or diagnosis of oral cancer [65]. In a similar study, a Cu@Ag/CNF-based NBS was designed to enhance the Raman spectroscopic through the interaction of a strong electric field as well as the chemical enhancement effect in order to achieve a highly sensitive detection of oral cancer biomarkers, such as nitrates, nitrites, thiocyanates, proteins, and micromolar-concentration amino acids in saliva samples. In particular, Navami et al. also combined the random forest algorithm for classification, which produced a robust classification accuracy of 87.5 %, specificity of 92 %, and sensitivity of 88 %, providing a new idea for early noninvasive oral cancer diagnosis [66]. Liu et al. integrated plasma Ag nanocubes (AgNC) into a microfluidic chip to enhance Raman spectroscopy through its high electromagnetic field properties, and to enhance nucleic acid endonuclease activity by obtaining high surface temperatures through Au electrodes inside the AgNC, thus accurately detecting oral cancer-associated DNA in saliva and realizing early prediction of OSCC [67].

## 5 Microfluidics based oral cancer treatment strategy

**Fig. 7:**
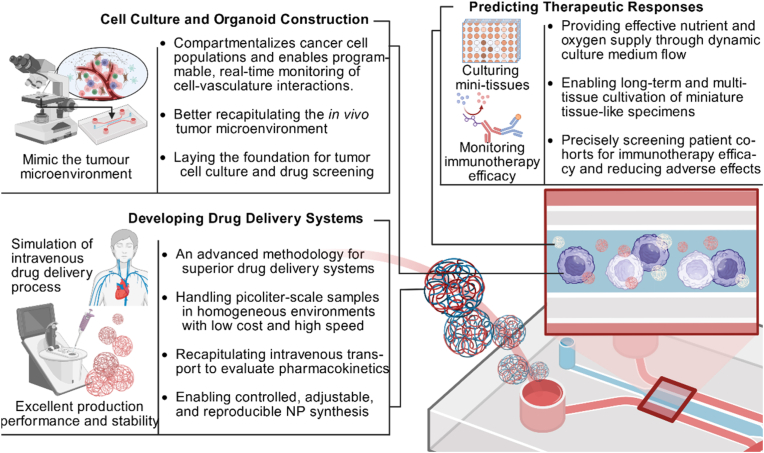
**Microfluidic technology empowers oral cancer treatment.** Microfluidics enables controlled drug synthesis with minimal batch variation, accurate prediction of treatment outcomes, and thorough assessment of drug safety and adverse reactions for oral cancer therapy. It integrates tissue culture platforms, precise nanomedicine fabrication, and personalized therapeutic screening to advance precision oncology.

Microfluidic systems provide a foundational platform for organ-on-a-chip models. While this technology may not directly treat oral cancer in patients, it offers critical insights into disease mechanisms and therapeutic strategies. Furthermore, cell culture and organ-on-a-chip-based approaches can inform drug development and efficacy testing (Fig. 7).

### 5.1 Cell culture and organoid construction

Although the utilization of microfluidic devices for cell culture or organoid construction is not directly relevant to the treatment of oral cancer, it may aid in the process of drug screening or drug side effect discovery. Microfluidic platforms are microfabrication devices featuring interconnected chambers, membranes, and grooves that manipulate a small amount of fluid [68]. Most of microfluidic platforms are predominantly fabricated via photolithography and surface micromachining techniques, utilizing polydimethylsiloxane, silicon, glass, polycarbonate, and polymethyl methacrylate (PMMA) as primary materials. Flow control mechanisms within these devices can be achieved through passive or active methods. The platform supports the simultaneous compartmentalization of multiple cancer cell populations by constant media flushing, which would allow programmed control and real-time monitoring of cancer cell-vessel system interactions through the platform's interconnected cell compartments [41,69]. Since the majority of therapeutics are administered intravenously, dynamically perfusing through vascular networks to reach tumor vasculature and extravascular tissues, the dynamic flow characteristics of microfluidic devices can recapitulate the transport processes of intravenous systemic therapy. This enables more precise and controllable evaluation of pharmacokinetics. Microfluidic devices have great potential for tumor cell culture and drug screening [70,71]. However, there are limitations in the design scale of existing microfluidic devices, and while it is a powerful tool for studying cancer-immune cell interactions, it is not applicable to high-throughput drug screening [72].

### 5.2 Employment of microfluidic devices to produce NPs

The limitations associated with conventional preparation methods are critical factors hindering the translation of NPs from research to clinical applications. Microfluidic technology offers exciting opportunities for developing drug delivery systems that can be engineered in a simple, cost-effective, and reproducible manner, positioning it as an advanced methodology for creating drug delivery system with well-defined physicochemical properties and batch-to-batch consistency [73]. The unique features of microfluidics lie in its capability to handle picoliter-scale (or smaller) samples, enable rapid mixing and mass transfer, precisely control reaction conditions and reagent addition, and achieve high cost-efficiency with shortened production timelines. More importantly, NPs can be reproducibly synthesized in microfluidic devices with well-controlled and tunable properties, such as size, shape, surface properties, and structure. The ability of microfluidic strategies to provide homogeneous reaction environment as well as rapid mixing of reagents, continuous adjustment of reaction conditions, and control of the order of mixing of reagents during the reaction makes them ideal for NPs synthesis in drug delivery applications [74]. The successful preparation of mRNA vaccines using microfluidics represents an important milestone in the use of microfluidic NPs for pharmaceutical applications, and it demonstrates the feasibility of using microfluidics for rapid scale-up of industrial-scale manufacturing [75]. Rao et al. found that the use of ultrasonication and mechanical extrusion for the preparation of cell-membrane-encapsulated NPs suffered from the potential to damage the NPs core and excessive energy loss, and therefore introduced microfluidic devices for Electroporation, in which electrical pulses between two electrodes can effectively promote the entry of NPs into cell membranes, shows good preparation efficacy and has the potential for mass and batch production. These NPs significantly improved magnetic resonance imaging (MRI) imaging and photothermal therapy (PTT) of oral cancer, and positively impacted the diagnosis and treatment of OSCC models [76].

### 5.3 Microfluidics for drug screening

Given the heterogeneity of OSCC and the need for more reliable and precise treatment strategies, the integration of microfluidic chip platforms has emerged as a potential way to improve the effectiveness of therapeutic interventions in oral cancer, utilizing these platforms to predict therapeutic response, ultimately contributing to more efficient applications in oral cancer management [77]. Millimeter-sized tissues for tumor biopsies often raise challenges for complex tissue preservation during long-term culturing, while advances in microfluidic platforms have made it possible to provide efficient nutrient and oxygen supply through dynamic media flow, enabling long-term culturing of tiny tissue samples. In addition, the segregation of different inter-compartments and tandem fluid flow through the microfluidic chip has made it possible to culture multiple tissues simultaneously and to move further towards tumor models that more closely mimic the *in vivo* TME environment. Furthermore, the dynamic flow characteristics of microfluidic devices can be analogous to the whole-body transport of intravenously administered medications, and help to assess the pharmacokinetics of the drugs in a more precise and controlled manner [70].

Viraj et al. developed a 3D-printed, mold-based microfluidic chip with serpentine channels and flat-bottomed microwells to dynamically culture personalized oral cancer spheroids, enabling high-throughput screening of seven combinations of three drugs (Paclitaxel, 5-Fluorouracil, and Cisplatin) against chemotherapy-resistant oral cancer stem cells and maintained a high level of tissue viability over a five-day period, and the clinical relevance of the drug screening data obtained in this study show significant advances over personalized sphere-based microfluidic devices [78]. Lei et al. encapsulated oral cancer cells (OEC-M1) in 3D agarose scaffolds and cultured in microchambers under perfusion conditions, thus providing a microenvironment with more specific exophysiological relevance to better mimic the complex *in vivo* microenvironment, and they embedded a pair of vertical electrodes on opposite side walls of the culture chamber for in situ impedance measurements, by which cell proliferation and chemosensitivity in the 3D cell culture form were monitored [79]. In addition to predicting chemosensitivity, it has also been shown that patient-derived OSCC tissues were embedded in a Myogel/fibrin *in vitro* environment and these tissues were loaded in a 3D microfluidic culture system. This study measured the efficacy of two immunotherapeutic agents, *anti*-PD-L1 and IDO-1 inhibitors [80], which will provide new ideas to accurately screen for populations that will benefit from immunotherapy, and potentially reduce the number of unnecessary side effects of treatment.

## 6 Conclusion and prospects

**Fig. 8:**
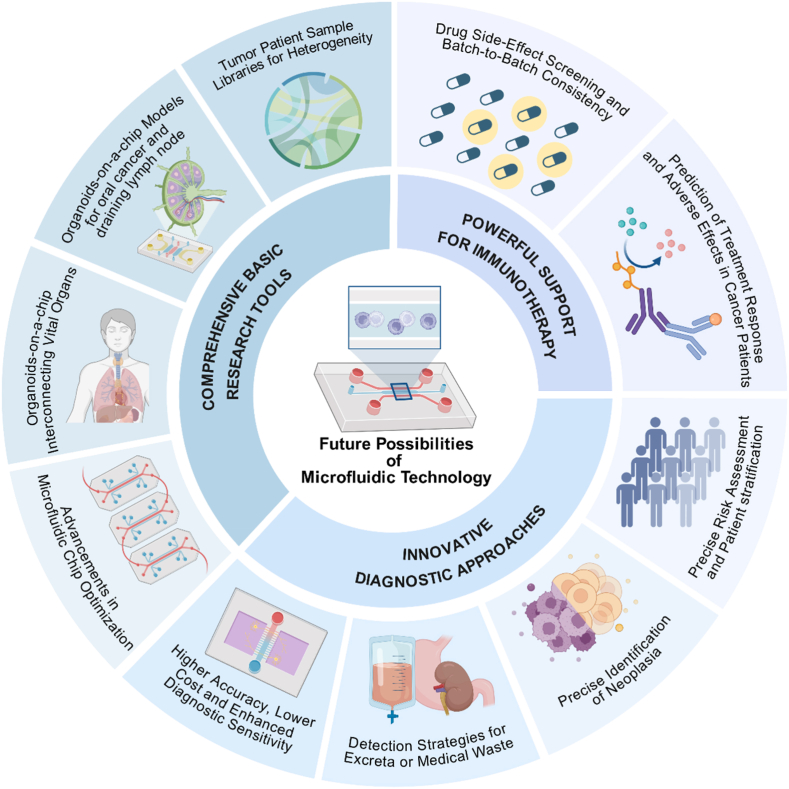
**Microfluidic technology holds great promise for the research and management of oral cancer.** Microfluidic technology may better support oral cancer research, offer more accurate diagnostic methods for oral cancer and precancerous lesions and enhance personalized predictions for oral cancer patients and related drug production efficiency.

Microfluidics is now widely used in the fields of basic research, diagnosis, and treatment of oral cancer, facilitating advances in early diagnosis and treatment strategies, and significantly improving clinical care for oral cancer patients. Liquid biopsy based on microfluidic strategies notably improves the early diagnosis of oral cancer and may predict the metastasis and treatment outcome of oral cancer, which enriches the field of liquid biopsy, and more accurate and economical microchip or NBS still need to be further explored and developed. Microfluidics-based cell culture modes or organoids-on-a-chip provide culture modes closer to the *in vivo* microenvironment and homeostasis, providing new ideas for drug screening, therapeutic efficacy predicting and even side effects combating [81]. Microfluidics with its high stability has also become an important technology for the production of NPs for the treatment of oral cancer and reduces the drug instability and cost caused by the differences between its production batches. In conclusion, microfluidics holds significant potential to enhance early diagnosis and treatment strategies for oral cancer. As an emerging paradigm in cell culture, this technology may serve as a crucial link between basic scientific research and clinical applications (Fig. 8).

### 6.1 Advanced design and fabrication of microfluidic devices

The design of microfluidic systems must align closely with genuine clinical needs, with strategic priorities including continuous enhancement of analytical sensitivity and specificity, user-friendliness, cost-effectiveness, and operational simplicity. However, the clinical translation of microfluidic technologies has been hindered by their reliance on peripheral equipment for sample preparation, signal detection, and readout, particularly limiting their implementation in resource-limited settings and low- and middle-income countries. In reality, many published advances remain at the proof-of-concept stage, where evaluations often utilize standardized reagents rather than authentic clinical specimens, thereby overlooking the inherent complexity of real-world samples and the associated processing and analytical challenges [82]. Furthermore, the integration of novel microfluidic platforms into clinical practice requires adherence to regulatory, legal, and ethical standards, necessitating close collaboration among regulatory bodies, healthcare providers, and technology developers. Targeted training and practical guidance are essential to lower barriers to clinical adoption, especially in regions with constrained health resources and under-resourced public health systems.

### 6.2 Enhancing oral cancer research through microfluidic platforms

Although the great potential of microfluidics for oral cancer tissue culture and organoids has been explored, the heterogeneity of oral cancers remains a major impediment to the exploration of cancer mechanisms and therapeutic strategies. Although organoids may be an important advancement compared to mouse models, organoids derived from cell lines or tissue models are still difficult to mimic the TME of patients given their differences. However, when patient-derived tissues are used to construct organoids or tumor cell culture models, inter-patient heterogeneity again interferes with mechanistic studies of tumor progression, and conclusions drawn may lose generalizability due to heterogeneity. The establishment of an organ microarray sample bank of oral cancer patients may be a promising strategy, as the large sample size of the bank can balance the differences in research conclusions due to inter-patient heterogeneity, and at the same time save research resources by generalizing and co-studying the same type of patients. Moreover, compared with the mouse model of patient-derived tumor xenograft, this strategy meets the requirements of scientific ethics and reduces the loss of experimental animals. Of course, innovating microfluidic strategies to improve the performance of organoids is still an important issue in the scientific community. The use of multiple microfluidic chips in tandem may better mimic the real systemic scenario of oral cancer patients, which has been demonstrated in the rest of the cancers, but not yet realized in the field of oral cancer research. LNM is still one of the most important basic and clinical research questions in the field of oral cancer research, and the establishment of the microfluidic technology better simulates the environment within the lymphatic system, especially the flow of lymphatic fluid may bring tumor-derived pro-metastatic signaling molecules produced by the primary tumor to the lymph nodes, which may represent the “preparation” stage of tumor metastasis. Secondly, we have shown in our previous study that LNM is not the end point of oral cancer progression, but LNM affects systemic tumor immunity, suppresses anti-tumor immunity in other organs, and ultimately promotes distant metastasis, resulting in the death of the patient [7]. In addition to constructing lymph node organ chips, major organ chips in the body such as liver, kidney, lung, bone, etc. Can also be connected in tandem, which may mimic the distant metastatic target organs of oral cancer and simulate the process of distant metastasis of oral cancer. In addition to this, other organs such as heart and spleen can also be connected in tandem with the organ chip, which may provide a reference for subsequent drug screening and predicting drug side effects.

### 6.3 Improving early detection and disease monitoring

We mentioned previously that microfluidics-based liquid biopsy strategies have improved the early diagnosis of oral cancer and the detection of oral cancerous lesions, and the development of microchip and NBS continues to be impressive, but technological innovations are necessary, and liquid biopsies require strategies that are more accurate, with a lower false-positive rate, lower diagnostic thresholds, and better economic feasibility. In addition to this, our previous studies investigated in developing PDFs previously considered as medical waste as an effective vehicle for liquid biopsy, “turning waste into treasure”, such a strategy emphasizes the need to reduce the depletion of normal body fluids and to increase the use of fecal or medical waste for testing, i.e., the development of a new medium for non-invasive early oral cancer prediction [51]. Differential diagnosis of oral cancer and precancerous lesions is also a key issue in clinical practice; precancerous lesions are not necessarily all cancerous and the transformation from precancerous to cancerous is a continuum for which it is difficult to find a cut-off point; liquid biopsy strategies may be able to indicate high-risk precancerous lesions and intervene with more aggressive strategies. Another interesting issue is the neck management strategy for early oral cancer, which we addressed in our previous study that cT1-2N0 OSCC patients with less than 30 % were confirmed to have LNM in postoperative pathology, and patients without LNM may not benefit from neck dissection, and the complications of this surgical procedure, such as shoulder dysfunction, will seriously affect the patient's life quality. Using microfluidic-based liquid biopsy strategies to accurately stratify cT1-2N0 OSCC patients, such as microchip for biomarkers in saliva or tissue exudate, to assess the risk level of LNM, and adopting different levels of cervical surgical interventions according to the risk level of different subgroups may result in a win-win situation in terms of both treatment and function.

### 6.4 Innovative strategies for therapeutic development and production

Immunotherapy has become an emerging and major strategy in the treatment of oral cancer, significantly improving the treatment landscape and enhancing the survival of oral cancer patients. The most important problem hindering the clinical promotion of immunotherapy is the low response rate of OSCC patients to immunotherapy. Screening immunotherapy-related biomarkers by microchip or NBS strategies may be able to screen patient groups with high response to immunotherapy or personalize the prediction of patients' response to immunotherapy. Moreover, by connecting the microfluidic-based oral cancer chip in series with multiple organ chips, the fluid in the microfluidic system better mimics the intravenous drug delivery mode in clinical practice, which is also helpful in predicting drug side effects. Second, nanomedicine-based immunotherapies have become an emerging theme in recent years, and microfluidics reduces the variation between batches of NPs production with its high stability, which may ameliorate the differences in drug effects and differences in toxicities due to differences in production processes or batch processes, and enables more economical production of nanomedicines, which may ultimately reduce the cost of drug manufacturing and alleviate the public health burden on low and middle income countries, and will play a positive role in clinical care and improved prognosis for oral cancer patients.

## CRediT authorship contribution statement

**Zi-Zhan Li:** Writing – review & editing, Writing – original draft, Methodology, Investigation. **Li-Ya Wei:** Writing – review & editing, Writing – original draft, Investigation, Conceptualization. **Lei-Ming Cao:** Writing – review & editing, Writing – original draft, Methodology, Investigation, Conceptualization. **Guang-Rui Wang:** Writing – review & editing, Writing – original draft, Investigation. **Han-Yue Luo:** Writing – original draft, Investigation. **Kan Zhou:** Writing – original draft, Investigation. **Xing-Zhong Zhao:** Writing – original draft, Investigation. **Bing Liu:** Writing – review & editing, Writing – original draft, Supervision, Investigation, Conceptualization. **Ming-Xue Zheng:** Writing – review & editing, Writing – original draft, Conceptualization. **Chun Xu:** Writing – review & editing, Supervision, Investigation. **Bo Cai:** Writing – review & editing, Writing – original draft, Conceptualization. **Lin-Lin Bu:** Writing – review & editing, Writing – original draft, Supervision, Methodology, Investigation, Funding acquisition, Conceptualization.

## Ethic approval

Not applicable.

## Declaration of competing interest

The authors declare that they have no known competing financial interests or personal relationships that could have appeared to influence the work reported in this paper.

## Data Availability

No data was used for the research described in the article.
